# Lipopolysaccharide treatment induces genome-wide pre-mRNA splicing pattern changes in mouse bone marrow stromal stem cells

**DOI:** 10.1186/s12864-016-2898-5

**Published:** 2016-08-22

**Authors:** Ao Zhou, Meng Li, Bo He, Weixing Feng, Fei Huang, Bing Xu, A. Keith Dunker, Curt Balch, Baiyan Li, Yunlong Liu, Yue Wang

**Affiliations:** 1 Center for Computational Biology and Bioinformatics, Indiana University School of Medicine, Indianapolis, IN 46202 USA; 2 Bioinformatics Program, Indiana University School of Informatics, Indianapolis, IN 46202 USA; 3 College of Automation, Harbin Engineering University, Harbin, Heilongjiang China; 4 Department of Medical and Molecular Biology, Indiana University School of Medicine, Indianapolis, IN 46202 USA; 5 Bioscience Advising, Indianapolis, IN 46227 USA; 6 Department of Pharmacology, Harbin Medical University, Harbin, Heilongjiang China

**Keywords:** Alternative splicing, Lipopolysaccharide, Mesenchymal stem cells

## Abstract

**Background:**

Lipopolysaccharide (LPS) is a gram-negative bacterial antigen that triggers a series of cellular responses. LPS pre-conditioning was previously shown to improve the therapeutic efficacy of bone marrow stromal cells/bone-marrow derived mesenchymal stem cells (BMSCs) for repairing ischemic, injured tissue.

**Results:**

In this study, we systematically evaluated the effects of LPS treatment on genome-wide splicing pattern changes in mouse BMSCs by comparing transcriptome sequencing data from control vs. LPS-treated samples, revealing 197 exons whose BMSC splicing patterns were altered by LPS. Functional analysis of these alternatively spliced genes demonstrated significant enrichment of phosphoproteins, zinc finger proteins, and proteins undergoing acetylation. Additional bioinformatics analysis strongly suggest that LPS-induced alternatively spliced exons could have major effects on protein functions by disrupting key protein functional domains, protein-protein interactions, and post-translational modifications.

**Conclusion:**

Although it is still to be determined whether such proteome modifications improve BMSC therapeutic efficacy, our comprehensive splicing characterizations provide greater understanding of the intracellular mechanisms that underlie the therapeutic potential of BMSCs.

**Electronic supplementary material:**

The online version of this article (doi:10.1186/s12864-016-2898-5) contains supplementary material, which is available to authorized users.

## Background

Alternative splicing (AS) is important for gene regulation and is a major source of proteome diversity in mammals [1] through altering the composition of mRNA transcripts by including or excluding specific exons [2]. AS can further modulate organism complexity not only by effectively increasing regulatory and signaling network complexity, but also by doing so in a temporal- and spatial-specific manner, supporting cell differentiation, developmental pathways, and other processes associated with multicellular organisms. Indeed, AS shows a strong relationship with organism complexity, as estimated by the organism’s number of different cell types [3]. The recent ENCODE Project concluded that at least 90 % of human genes express multiple mRNAs through alternative splicing of exons or exon segments [4]. As might be expected, deregulation of this process is associated with numerous diseases [5–10].

Bone marrow-derived mesenchymal stem cells (BMSCs) are adult stem cells capable of self-renewal and differentiation into numerous cell lineages, including osteocytes, adipocytes, and chondrocytes [11]. One promising use of BMSCs is repair of ischemia-damaged cardiac tissue. BMSCs are easy to expand in vitro, can be genetically modified and exhibit significant immunotolerance properties [12–14], making BMSCs an attractive candidate for tissue repair/regeneration therapy. Intramyocardial injection of BMSCs reduces inflammation, fibrosis, infarct size, ventricular remodeling, and therefore, improves cardiac function following tissue insult [15–18].

Because the majority of BMSCs are soon lost during after injection, the observed therapeutic effects likely derive from paracrine effects of bioactive molecules released from these cells [15, 16]. Indeed, BMSC-mediated release of cytoprotective protein factors or transfer of intracellular components (e.g.,mRNAs, microRNAs, and proteins) via cell membrane exosomes, represents a novel mechanism of cell-to-cell communication [19]. To date, however, clinical trials have demonstrated that while effective, delivery of BMSCs to ischemic myocardium results in only modest and short-lived benefits [20, 21]. Therefore, there is a critical need to elucidate the mechanisms by which BMSCs mediate their therapeutic benefits, including identification of their specific paracrine factor(s), and conditions under which their functions can be optimized.

Upon injection into damaged heart tissue, BMSCs face a hypoxic, ischemic environment that severely limits their therapeutic efficacy. Thus, preconditioning BMSCs with various growth factors and endogenous or exogenous molecules has been used to improve BMSC therapeutic efficacy [22–24]. Indeed, it has been reported previously that bacterial endotoxin (lipopolysaccharide, LPS) could stimulate BMSCs to release paracrine factors, including angiogenic growth factors, cytokines, and chemokines that facilitate tissue repair [13, 14]. In addition, our previous study suggested that BMSC expression of the LPS receptor, toll-like receptor 4 (TLR4), regulates BMSC paracrine properties and intracellular STAT3 signaling cascades [25]. Moreover, preconditioning of BMSCs with LPS improves their therapeutic efficacy in rodent models of ischemia/reperfusion injury [23]. However, BMSC transcriptomic changes (in particular, alterations in mRNA transcript processing and splicing) that occur following LPS stimulation have been little studied.

Besides use as an attractive therapeutic tool for repairing ischemic heart, BMSCs have been used for numerous other diseases, including graft-versus-host disease, Crohn’s disease, stroke, cartilage defects, diabetes and many others [26–31]. With the growing incidence of bacterial endotoxin LPS detected in older or immunocompromised patients with multiple-drug resistant bacteria, diabetes, cancer, indwelling IV catheters, and on complex chemotherapy regimens [32, 33], it is of great importance to study whether the stimulation of these implanted BMSCs by endogenous LPS would alter their therapeutic efficacy. Moreover, because MSCs are present in bone marrow and many other tissues, it merits extensive investigation whether LPS stimulation of these endogenous MSCs would influence the clinical outcomes of complex therapeutic regimens.

Despite BMSC’s strong clinical potential, the role(s) of alternative splicing in LPS response has not been fully explored. The recent development of high-throughput sequencing technology has now made transcriptome-wide profiling of splicing isoforms possible. In this study, we used RNA-seq analysis of BMSCs to identify and characterize gene transcripts whose splicing patterns were altered by LPS treatment.

## Results

To investigate LPS-induced transcriptomic changes in BMSCs due to alternative splicing, RNA-seq analysis was conducted on BMSCs before and after LPS treatment, in triplicate. A strand-directed single-end RNA-seq protocol (75 bp reads) was used with the SOLiD 5500xl instrument.

The total analysis resulted in 326 million reads, with each of the six samples ranging from 43 to 59 million reads. After removing the reads with low sequencing quality (see Methods) and filtering reads mapped to ribosomal RNAs and repeats, the remaining reads were mapped to the standard mouse reference genome (mm9). The total number of mappable reads in each sample ranged from 29 to 36 million, with an average mapping percentage of 59 %. Among the mappable reads in each sample, 3.8 to 5.0 million are mapped to protein coding exons, and 2.8 to 4.0 million are mapped to splice junctions. Detailed mapping statistics for the six samples are listed in Additional file 1.

### LPS-induced alternative splicing

We applied a MISO (Mixture of Isoform) algorithm [34] to identify alternative splicing events elicited by LPS treatment. Based on a Bayesian inference framework, MISO is a probabilistic framework that quantitates the expression levels of alternatively spliced genes from RNA-Seq data, and identifies differentially regulated exons across samples. MISO computes Percent Spliced In (PSI, or Ψ) values for each alternative splicing event, representing the fraction of a gene’s mRNA that includes the exon. For each event, MISO also calculates a Bayesian Factor (BF) that quantifies the likelihood of the changes. For instance, [BF] = 5 indicates it is five times more likely that a specific alternative splicing event occurred than did not occur.

**Fig. 1:**
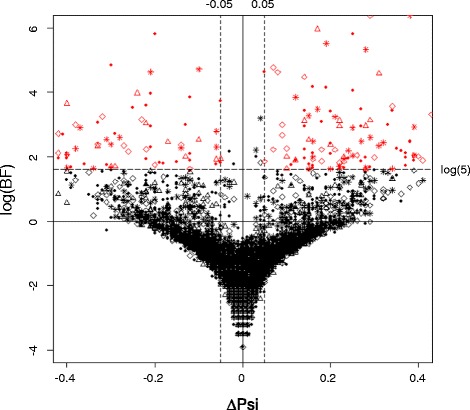
LPS-induced alternative splicing events. Scatter plot of all the AS events identified in MISO. The X-axis represents ∆Ψ, and the Y-axis represents log (BF). The shape of the dots indicates the type of the events. Specifically, circle indicates cassette exon events; star indicates intron retention events; triangle indicates alternative 5′ splice site events; and diamond indicates alternative 3′ splice site events. Alternatively spliced events with BF ≥ 5 are colored in red

**Fig. 2:**
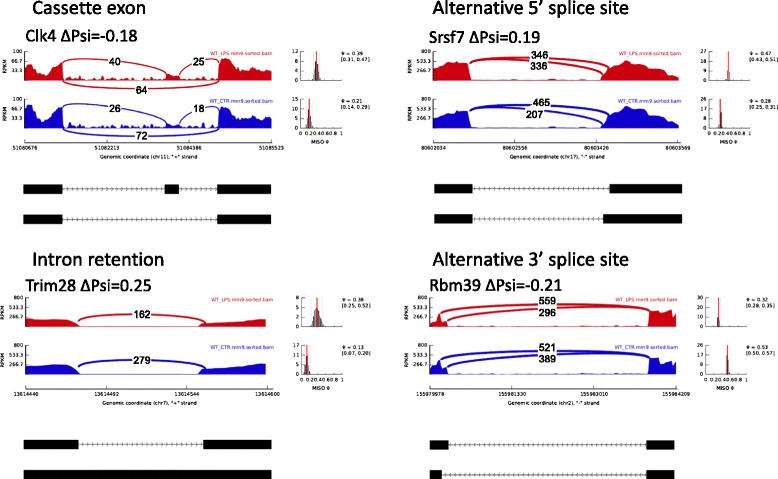
Sashimi plots of four types of AS events. Sashimi plots of four types of AS events were shown, including cassette exon, intron retention, alternative 5′ and 3′ splice site. The red plots represent the LPS treated condition, and the blue ones represent controls. The X-axes indicate genomic locations, and the Y-axes indicate transcription intensity. In each plot, a “sashimi-like” region indicates a heavily transcribed region, in this case, exonic region. The blank regions between exonic regions indicate intronic regions. The “bridges” crossing exons indicate junction reads. The numbers of junction reads are shown on the “bridges”. The exonic structure of each AS event is shown below each Sashimi plot. On the right it displays the estimated Ψ (red line) value and the full posterior distribution (black bars)

Overall, we identified 197 exons whose splicing patterns differed between control and LPS-treated BMSCs (Bayesian factor [BF] > 5 and |ΔΨ| > 0.05). This number represents 2.32 % of all 8,475 events whose inclusion percentages could be reliably measured from the RNA-seq data; these genes generally had higher expression levels to generate enough read depth for splicing analysis. For genes with lower expression levels, our RNA-seq experiment did not have enough read depth for such analysis. The 197 LPS-induced alternatively spliced events included 82 cassette exons, 28 alternative donor site events (5′-ss), 45 alternative acceptor site events (3′-ss), and 42 intron retention events. Figure 1 demonstrates the magnitude (X-axis) and significance (Y-axis) of LPS-induced splicing pattern changes on all the alternatively exons that could be reliably identified by MISO under both untreated and LPS-treated conditions (Fig. 1). Among these 197 events (red dots in Fig. 1), 117 showed positive ∆Ψ values, indicating that the percentage of transcripts containing the specific exon increased in the LPS-treated samples compared to control samples. Similarly, 80 events showed negative ∆Ψ values, indicating a decrease in the percentage of transcripts containing specific exons. For each of the four types of splicing events (cassette exons, alternative 5′-donor sites, alternative 3′-acceptor sites, and intron retention), we show one Sashimi plot for the exons with the largest LPS-induced changes (either increases or decreases) in percentage of inclusion in the gene product (Fig. 2). The Sashimi plot demonstrates the RNA-seq read densities along exons and junctions, in the context of the structure of the gene’s isoforms. In addition, the distribution and the confidence intervals of the estimated Ψ under both conditions (LPS vs. untreated) are also included.

**Fig. 3:**
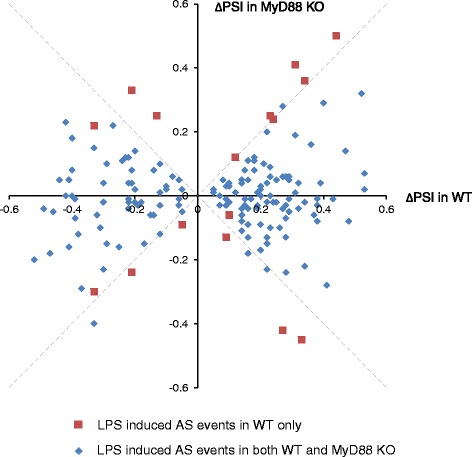
LPS-induced splicing changes in wild-type BMSC were repressed in MyD88−/− cells. The X-axis and Y-axis represents ∆PSI in wild type and MyD88 knock out animals respectively. Blue diamond represents LPS induced AS events in wild type only, and red square represents LPS induced AS events in both wild type and MyD88 knock-out cells

To validate whether the alternative splicing events were induced by LPS treatment, we performed RNA-seq on BMSCs derived from MyD88^−/−^ animals before and after LPS treatment. MyD88 is a key signaling molecule responsible for LPS response [35]. Among the 197 LPS-induced alternative splicing events in wild-type BMSCs, 189 did not occur following LPS treatment of MyD88^−/−^ BMSCs (Fig. 3). This observation indicates that a large majority of BMSC splicing changes were a direct consequence of LPS induction, and such effects were negated in cells whose LPS response is compromised. It should be noted that in addition to MyD88 pathways, LPS also functions through TRIF pathways [36]; the functions of TRIF pathway is intact in the MyD88^−/−^ cells. This partially explains why some LPS-induced splicing effects remained in MyD88-deficient animals.

Among the 197 LPS-induced alternative splicing events, 103 were located in the coding regions of transcripts, and 94 were either in the 5′- or 3′- untranslated regions (UTRs). Among the 103 alternatively spliced coding events, 65 were composed of multiples of three nucleotides, leading to the inclusion or exclusion of specific amino-acid residues in the final protein products. These events could potentially generate multiple viable protein products having the same translation frame. Thirty-eight of the 103 coding exons contained either a premature stop codon, and/or a shift in their translation frames. Such events trigger either nonsense-mediated decay (NMD) mechanisms [37], or a translated protein having a complete different amino acid sequence downstream of the alternatively spliced exon.

**Fig. 4:**
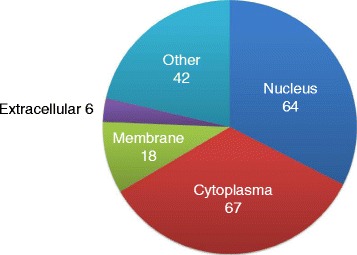
Distribution of AS genes in different cellular locations

We then systematically examined the localization and functions of the gene products possessing alternatively spliced exons (Fig. 4 and Additional file 2). Among them, 64 were nuclear proteins, including 17 transcription regulators, 13 enzymes, 2 kinases, 1 peptidase, and 1 ligand-dependent nuclear receptor. The 67 cytoplasmic alternatively spliced gene products included 12 enzymes, 9 kinases, 6 transporters, 3 peptidases, and 2 translation regulators. In addition, we also observed six potentially secreted proteins and 18 plasma membrane-spanning proteins. A detailed list of the genes in each category is provided in Additional file 3. These results strongly suggest that LPS induces splicing changes in highly diverse proteins having a variety of cellular functions.

To understand the biological functions of genes whose splicing patterns were altered by LPS treatment, we conducted functional annotation analysis using the Database for Annotation, Visualization and Integrated Discovery (DAVID) v6.7 [38]. Three functional terms in the SP_PIR (Swiss-Prot and Protein Information Resources) category showed significant enrichment in our gene list. Among the 161 genes that could be mapped to DAVID gene annotations, 97 categorized as phosphoproteins (*p*-value = 7.2×10^−12^, FDR = 6.6×10^−10^). In addition, 26 genes contained zinc finger domain proteins (*p*-value = 3.6×10^−5^, FDR = 2.2×10^−3^) whose functions range from DNA or RNA binding to protein-protein interactions and membrane association [39]. Furthermore, 35 genes were involved in protein acetylation (*p*-value = 1.3 ×10^−3^, FDR = 3.2×10^−2^). Together, these results suggest that LPS treatment has major effects on the splicing patterns of signaling proteins.

Both gene expression levels and splicing patterns may be altered by BMSC responses to LPS treatment. While differential gene expression may lead to changes in the abundance of the entire gene product, alternative spicing modifies the structural composition of a specific protein. To evaluate to what extent the two mechanisms interact, we examined the number of genes present in both differentially expressed and alternatively spliced gene sets. We utilized edgeR [40] to identify genes differentially expressed between LPS-treated and control samples. In total, 416 differentially expressed genes were identified using a false discovery rate ≤ 0.05. Surprisingly, only one gene, *Plscr2* (Phospholipid Scramblase 2) was both differentially expressed and alternatively spliced. The expression level of *Plscr2* increased 1.77-fold in LPS-induced samples with FDR = 0.01, while the percentage of inclusion of one cassette exon in the 3′-untranslated region (3′-UTR) increased by 0.16.

### Known protein domains are alternatively spliced in LPS-induced transcripts

Alternatively spliced exons residing in known protein domains are more likely to disrupt protein function. Therefore, we systematically searched the overlap between LPS-induced AS events for known protein family domains documented in the pfam database [41]. Among 65 alternatively spliced exons that did not disrupt codon frame, seven overlapped with known protein domains (Table 1). In addition, seven other known domains that overlapped flanking exons had functions ranging from RNA and protein binding, enzymatic activities, methyltransferase activity, phosphopantetheinyl transferase activity, RNA editing, and microRNA processing.

**Table 1:** Alternatively spliced genes containing known protein domains

Gene Symbol	AS Type	Pfam Domain	Domain Description
Ttc13	cassete exon	TPR_11	TPR repeat
Rabep1	cassete exon	Rabaptin	Rabaptin
Camk1d	cassete exon	Pkinase	Protein kinase domain
Nr1h2	alternative 5′ splice site	Hormone_recep	Ligand-binding domain of nuclear hormone receptor
Adarb1	alternative 5′ splice site	A_deamin	Adenosine-deaminase (editase) domain
Scoc	alternative 3′ splice site	DUF2205	Predicted coiled-coil protein
Ppip5k2	alternative 3′ splice site	His_Phos_2	Histidine phosphatase superfamily (branch 2)

### Alternative splicing in known protein domains may affect protein-protein interactions

**Fig. 5:**
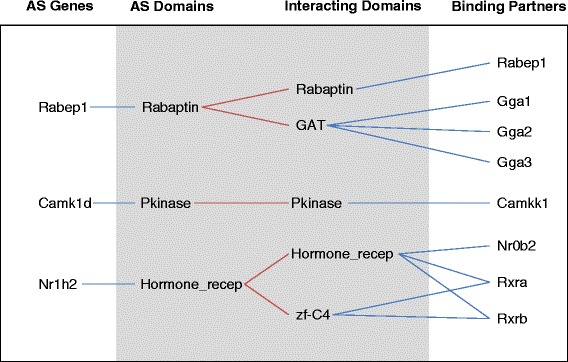
PPI with both structural and experimental evidences. Ten AS gene products involved in protein-protein interactions. Gene symbols are displayed in white regions, and corresponding protein domains are displayed with gray background. Blue line indicates a gene/protein contains a domain, and a red line indicates an interaction between protein domains

To examine whether alternatively spliced protein domains modulate protein-protein interactions, we searched for their binding partners based on two criteria: (1) at least one experimental study supporting direct interaction between the partner protein and the alternatively spliced protein in a known protein-protein interaction network [42, 43]; and (2) at least one structural study in the Protein Data Bank (PDB) supporting direct interaction between a domain in the binding partner and the domain modified by alternative splicing. For the first criterion, we merged two datasets of experimentally validated direct interactions [42, 43] and compiled a library of 9,795 protein-coding genes with 80,518 experimentally validated interactions. For the second criterion, we derived the domain interactions in PDB from iPfam [41] and then searched for proteins containing these domains in Pfam [41]. In total, 3,573 interactions with structural evidence were found between 13 alternatively spliced coding transcripts and 3103 binding partners. By joining two interaction tables, we identified eight interactions having both experimental and structural evidence. As shown in Fig. 5, these eight interactions involved three genes with altered splicing domains, Rabep1 (Rab GTPase-binding effector protein 1), Camk1d (Calcium/Calmodulin-Dependent Protein Kinase 1D), and Nr1h2 (nuclear receptor subfamily 1, group H, member 2). The alternatively spliced exons in these genes overlapped with known protein domains, including rabaptin, pkinase, and ligand-binding domain of nuclear hormone receptor.

The differences in the percentage of inclusion for these three events ranged from 14 to 31 %. The potential protein partners included Rabep1, Gga1 (Golgi-associated, gamma adaptin ear containing, ARF-binding protein 1), Gga2 (Golgi-associated, gamma adaptin ear containing, ARF binding protein 2), Gga3 (Golgi-associated, gamma adaptin ear containing, ARF binding protein 3), Camkk1 (calcium/calmodulin-dependent protein kinase kinase 1, alpha), Nr0b2 (nuclear receptor subfamily 0, group B, member 2), Rxra (retinoid X receptor, alpha), and Rxrb (retinoid X receptor, beta). LPS-induced splicing changes could significantly impact these proteins’ interactions with their partners. Among these putative protein interaction partners, only one protein, Nr0b2 (nuclear receptor subfamily 0, group B, member 2), was not expressed.

### Intrinsic disorder and molecular recognition features in LPS-induced alternative spliced regions

**Fig. 6:**
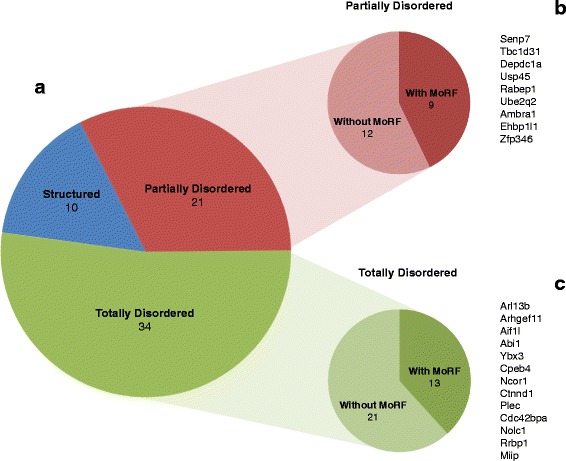
Predicted disorder of AS gene products. **a** The distribution of 65 non-frameshifting protein coding AS genes in three categories, including totally disordered, partially disordered and structured. **b** The MoRF containing and non-containing events among partially disordered AS genes. The list of MoRF containing genes is shown on the right. **c** The same distribution and gene list as panel B but it is for totally disordered AS genes

It was previously reported that alternatively spliced regions are enriched with unfolded protein regions (intrinsic disorder) [44]. To examine these features within LPS-induced alternatively spliced regions (cassette exons, alternative 5’/3′ exons and retained introns), we performed disorder prediction on the protein sequences of these regions using VSL2B [45], a bioinformatics algorithm for predicting intrinsically disordered regions based on the biophysical properties of amino acids. Among the alternative regions of 65 protein sequences translated from LPS-induced alternative splicing events, 34 (52.3 %) were predicted to be totally disordered, 21 (32.3 %) partially disordered, and only 10 (15.3 %) totally structured (Fig. 6). These percentages are consistent with previous reports that alternatively spliced exons tend to locate in intrinsically disordered regions [46].

A molecular recognition feature (MoRF) is a region in an RNA that undergoes a disorder–order transformation while bound by another protein. We predicted MoRF regions within the alternative regions using the software tool MoRF2 [44]. As a result, among the 55 alternatively spliced exons in the partial or totally disordered regions, 22 contained regions predicted to be MoRFs (Fig. 6, Additional file 4); these regions could thus be regarded as potential protein-protein interaction sites.

### Post-translational modification sites within alternatively spliced regions

**Fig. 7:**
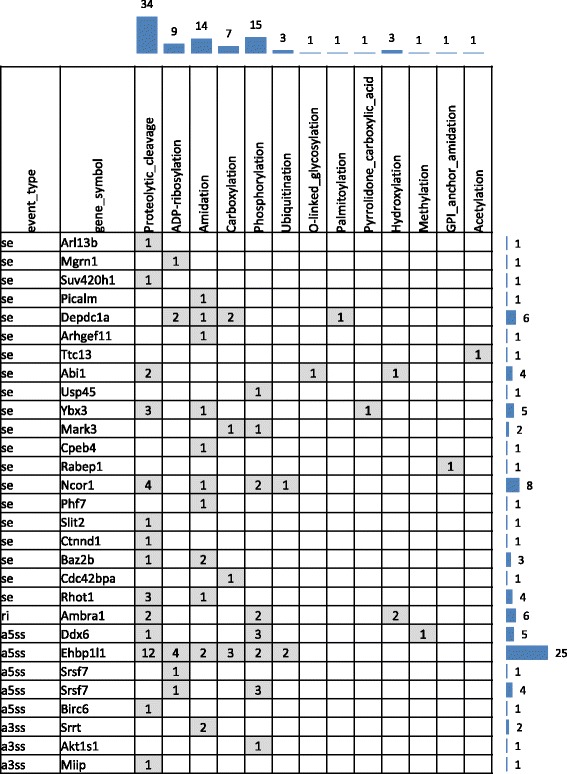
Predicted PTM sites in AS regions. Column displays different types of PTM sites, and row displays the event types and LPS-induced AS genes. The numbers in the shadowed grids on the crossing of gene A and PTM type B shows how many type B PTM sites fall in the AS region of gene A. The total number of PTM sites in each gene is displayed on the right, and the total number of PTM sites in each type is displayed on the top

We next annotated post-translational modification (PTM) sites in regions affected by LPS-induced alternative splicing. We searched known PTM sites deposited in UniProt, and we also predicted novel ones using ModPred [47]. Three alternatively spliced exons containing known PTM (phorphorylation) sites localized to three genes, Abi1 (abl-interactor 1), Depdc1a (DEP Domain-Containing 1), and Ybx3 (Y box-binding protein 3). In addition, 13 PTMs were predicted to occur in 29 alternatively spliced regions, including proteolytic cleavage, phosphorylation, amidation, hydroxylation, carboxylation, ADP-ribosylation, O-linked glycosylation, acetylation, GPI anchor amidation, palmitoylation, pyrrolidone carboxylic acid, methylation and ubiquitination (Fig. 7). Proteolytic cleavage sites were the most common PTM sites, appearing in 14 alternative regions. It is possible that LPS affects the signaling activities of these proteins by inclusion or exclusion of the PTM sites in the final protein product (i.e., whether or not it is cleaved).

### Characterization of potential splicing regulators

**Fig. 8:**
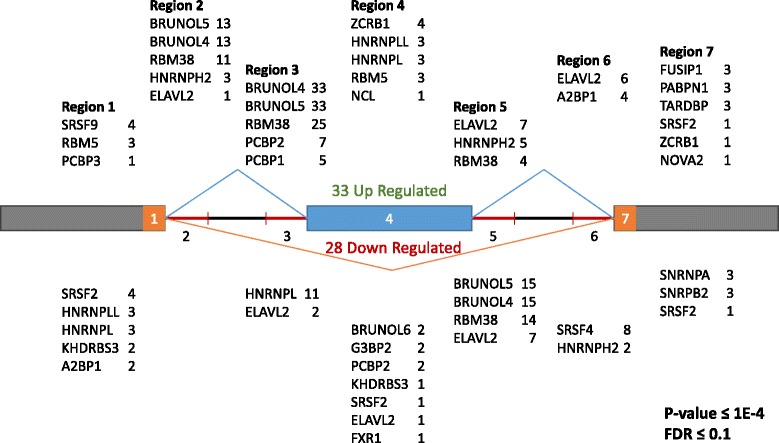
RNA binding protein (RBP) motifs in regulatory regions of differentially spliced events. RBP names and their occurrences are listed adjacent to corresponding regulatory regions

We defined 7 regulatory regions for each cassette exon event (Fig. 8), among which Region 1 and 7 are 150 nt constitute exon segments, Region 2, 3, 5 and 4 are 300 nt intronic segments, and Region 4 is the whole cassette exon. We used FIMO [48] to search for CISBP-RNA [49] motifs within the regulatory regions of both up-regulated and down-regulated cassette exon events. With *p*-value cutoff of 1E-4 and FDR cutoff of 0.1, we identified 29 RBP motifs in the up-regulated events, and 23 in the down-regulated events. BRUNOL5, BRUNOL4 and RBM38 are the most frequently observed RBPs. Their motifs concentrate in Region 2 and 3 for up-regulated events, and in Region 5 for down-regulated events. These three proteins are all known as RNA-splicing related. Motifs of several other RNA-splicing related proteins, including SRSF2, HNRNPL, HNRNPLL, HNRNPH2 and PCBP2, are observed in both up-regulated and down-regulated cassette exon regulatory regions. Some RBPs (SRSF9, RBM5, PCBP3, PCBP1, ZCRB1, NCL, FUSIP1, PABPN1, TARDBP and NOVA2) are found exclusively in up-regulated cassette exon events, and some (KHDRBS3, BRUNOL6, G3BP2, FXR1, SRSF4, SNRNPA, SNRPB2) are found exclusively in down-regulated events.

## Discussion

Lipopolysaccharide (LPS, endotoxin) is a complex associated with the outer membrane of Gram-negative bacteria, capable of triggering a series of cellular responses in many cell types. One promising advance is to use LPS as a pre-conditioning agent to improve BMSC therapeutic efficacy for repairing ischemic, injured tissues [23, 50]. For such application, because LPS is a potent stimulant for the host immune system, BMSCs should be washed using PBS to completely remove any residual endotoxin before administration. We reported previously that BMSCs treated with LPS produced more angiogenic factors VEGF, IGF-1 and HGF [51, 52] which can spur the formation of new blood vessels in ischemic tissue and survival and differentiation of implanted BMSCs. By contrast, with the growing incidence of sepsis, in which free LPS can bind to and activate Toll-like receptor 4 on many cell types, the roles of LPS on endogenous BMSCs and other cell types are worth detailed investigation.

Microarray studies have reported that expression levels of hundreds of genes can be altered after LPS treatment in different tissues. In recent years, high-throughput RNA sequencing technology has provided a more accurate and comprehensive measurement of RNA transcript levels and their isoforms than historic array-based methods. This technological advance has enabled measuring not only gene expression level alterations amongst different conditions, but also complicated splicing pattern changes in response to specific cellular perturbations. In this study, we systematically identified alternative splicing changes in mouse bone marrow-derived mesenchymal stem cells (BMSCs) in response to LPS treatment, using RNA-seq technology. We further implemented a series of bioinformatics tools to evaluate the biological functions of alternatively spliced exons and their host genes.

We observed strong enrichment in three functional categories amongst the gene products whose splicing patterns were altered by LPS treatment, phosphoproteins, zinc finger proteins, and proteins subject to acetylation. Most of these proteins were signaling proteins, and the subtle differences in their splicing isoforms could affect their function.

Among 161 gene products containing AS exons, 97 belonged to phosphoprotein families, five of which contained documented phosphorylation sites in their AS regions found in the UniProt database. These proteins included Kansl2 (KAT8 regulatory NSL complex subunit 2), Depdc1a (DEP domain-containing 1), Abi1 (abl-interactor 1), Ybx3 (Y box-binding protein 3), and UBl4a (Slc10a3-Ubl4 readthrough). The functions of these proteins strongly associate with the functions of BMSCs. For instance, Abi1 contains one cassette of exons whose percentage of inclusion increased by 14 % after LPS induction (ΔΨ = 0.14), with one phosphorylation site in the AS region documented in the UniProt database. Widely expressed with highest levels in bone marrow, spleen, brain, testes, and embryonic brain, Abi1 may negatively regulate cell growth and transformation by interacting with the nonreceptor tyrosine kinases ABL1 and/or ABL2, thus regulating EGF-induced Erk pathway activation and EGFR signaling. In addition to these five proteins, eight other AS regions were predicted to have phosphorylation sites, based on their amino acid contents. These proteins included Usp45 (ubiquitin-specific peptidase 45), Mark3 (MAP/microtubule affinity-regulating kinase 3), Ncor1 (nuclear receptor corepressor 1), Ctnnd1 (cadherin-associated protein, beta 1), Ambra1 (autophagy/beclin-1 regulator 1), Ddx6 (DEAD (Asp-Glu-Ala-Asp) box helicase 6), Ehbp1l1 (EH domain binding protein 1-like 1), and Akt1s1 (AKT1 substrate 1). Overall, LPS may affect the functions of these proteins by including/excluding specific domains amenable to phosphorylation.

Among the proteins containing LPS-induced alternative splicing events, 25 contained multiple types of zinc finger domains, including PHD (Plant Homeo Domain), RING (Really Interesting New Gene), and C2H2-type zinc-finger domains. Four proteins, Phf7 (PHD finger protein 7), Phf20 (PHD finger protein 20), Phf20l1 (PHD finger protein 20-like 1), and Phrf1 (PHD and ring finger domains 1), contained PHD-type zinc finger domains known to recognize trimethylated histone lysines (thus possibly influencing chromatin structure). Four other proteins, Rnf14 (ring finger protein 14), Rad18 (RAD18 homolog), Trim28 (tripartite motif-containing 28), and Trim2 (tripartite motif-containing 2), all contain RING-type zinc fingers, known ligases for ubiquitination enzymes and their substrates. It is well documented that both PHD and RING-type domains are usually involved in protein-protein binding [53, 54], and such binding could possibly be disrupted by splicing variations.

**Fig. 9:**
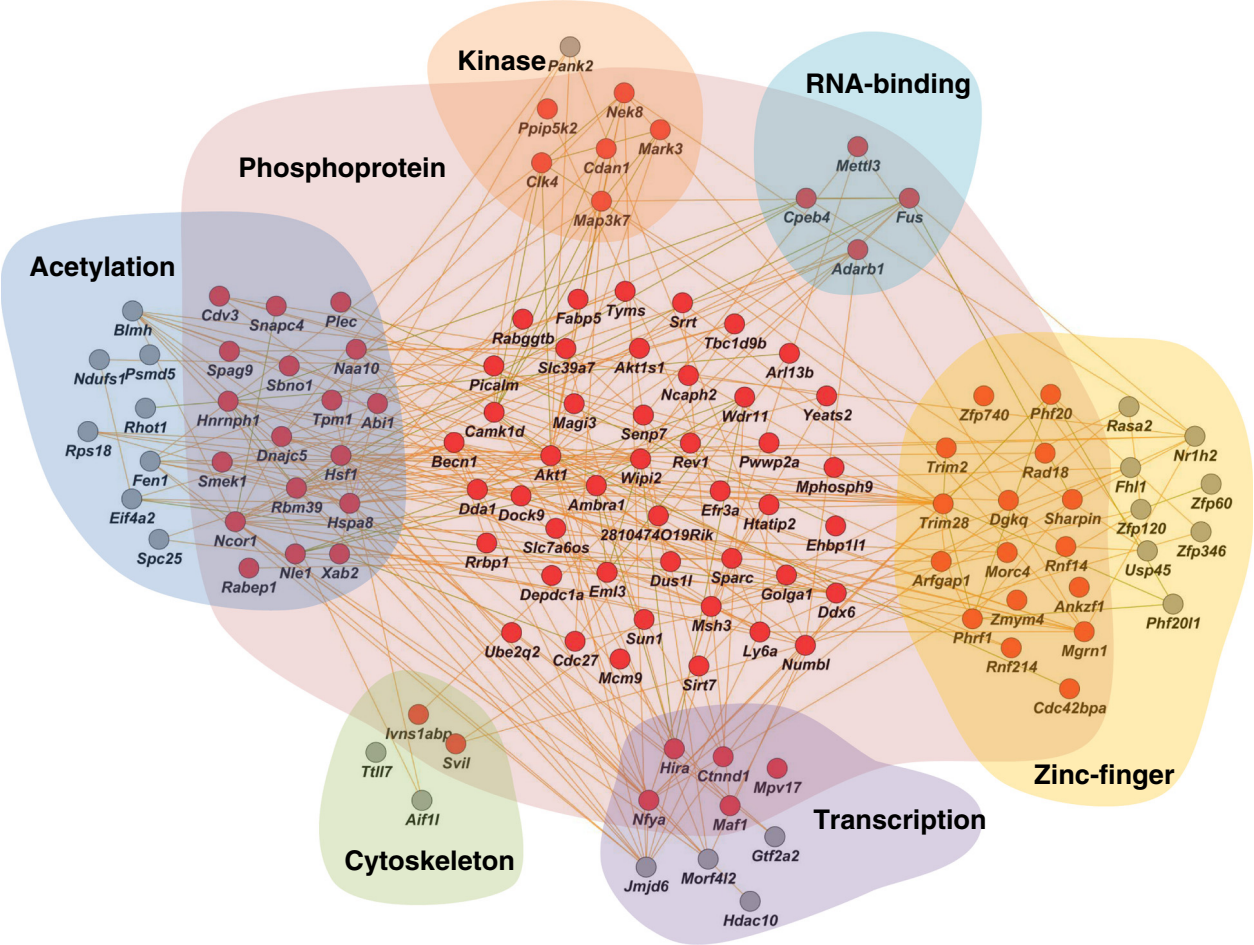
Predicted interaction network among LPS-induced AS genes. Red nodes indicate genes producing phosphoproteins, and gray nodes indicate genes not involved in protein phosphorylation. Genes associated with terms other than phosphoproteins are clustered in corresponding shadowed areas. These terms include acetylation, cytoskeleton, transcription, zinc-finger, RNA-binding and kinase

Overall, the LPS-induced AS genes could be classified into several categories (Fig. 9), including kinases, zinc-finger proteins, transcription, RNA-binding, cytoskeleton, and protein acetylation. Many of these proteins were also phosphoproteins, which play significant roles in cell signaling. Analysis of the relationship between splicing and protein structure has suggested that AS exons play major roles in controlling protein-protein interactions (PPIs) through disrupting either known protein interaction domains or molecular recognition sites, which typically locate in intrinsically disordered regions. Our analysis suggests that LPS-induced alternative splicing could affect PPIs through both mechanisms. In particular, protein interaction domains of three proteins with known PPI partners were disrupted by LPS-induced splicing alterations (Fig. 5). Interestingly, all three interactive domains could self-interact (forming domain-domain interactions with themselves), and one of these domains facilitates homodimerization of Rabep1 (RAB GTPase binding effector protein 1). Expressed in embryonic tissues and most types of stem cells, Rabep1 showed abundant expression in BMSCs (about 30 RPKM). Homo-dimerization of this protein is involved in early endosome fusion [55], an event directly related to the paracrine effects of BMSCs, where small vesicles are released when multivesicular endosomes fuse with the plasma membrane [56, 57]. In addition, Rabep1 also moderates intracellular transportation between lysosomes and the Golgi apparatus [58], and between the Golgi apparatus and endoplasmic reticulum [59]. LPS treatment also increased the inclusion of the interaction domain by 14 %, which could increase either homodimerization or heterodimerization with other interaction partners.

We further evaluated how differences in splicing patterns in transcriptional regulators affected their regulatory activity by assessing gene expression changes of their downstream target genes. NFYA (nuclear transcriptional factor Y) contains an alternative acceptor site whose splicing pattern in BMSCs is altered by LPS treatment; the overall percentage of inclusion of the alternative acceptor site decreased by 31 % (Sashimi plot for NFYA shown in Additional file 5). Moreover, the expression of five downstream target genes of NFYA were enriched for genes found differentially expressed (*p*-value ≤ 0.01) by LPS treatment (FDR ≤ 0.05), including COL11A1 (collagen, type XI, alpha 1), COL5A3 (collagen, type V, alpha 3), FGFR2 (Fibroblast Growth Factor Receptor 2), PGK1 (phosphoglycerate kinase 1) and RGS4 (regulator of G-protein signaling 4). It was previously reported that NFYA activates transcription levels of COL11A1 and FGFR2 [60]; these two genes were both downregulated by LPS, suggesting inhibition of NFYA function by the removal of 18 nt (or 6 amino acids) after LPS treatment, thus impacting NFYA downstream effectors.

## Conclusion

In summary, we used RNA sequencing to analyze LPS-induced alternative splicing changes in BMSCs. LPS modified the alternative splicing pattern of phosphoproteins, zinc finger proteins, and proteins subject to acetylation. Most of the affected proteins were signaling proteins that could change BMSC biological function. Although it is still to be determined whether such modifications underlie BMSC therapeutic efficacy, our characterizations provide greater understanding of the mechanisms and clinical usage of promising BMSC therapies.

## Methods

### Preparation of mouse BMSCs

A single-step stem cell purification method was employed as previously described [61]. Briefly, BMSCs were collected from the bilateral femurs and tibias of sacrificed mice by removing the epiphyses and flushing the shaft with complete media, Iscove’s Modified Dulbecco’s Medium (IMDM; Life Technologies) and 10 % fetal bovine serum (Life Technologies), using a syringe with a 26G needle. Cells were disaggregated by vigorous pipetting and passed through a 30-μm nylon mesh to remove any remaining clumps of tissue. Cells were then centrifuged for 5 min at 500 g at 24 °C. The cell pellet was then resuspended and cultured in 75 cm^2^ culture flasks in complete media at 37 °C with 5 % CO_2_. Since BMSCs preferentially attach to polystyrene [62], after 48 h, floating non-adherent cells were discarded. Fresh complete media was added and replaced every three or four days thereafter. When the cells reached 90 % confluence, MSC cultures were recovered by the addition of a solution of 0.25 % trypsin-EDTA (Invitrogen) and passaged. Cell passage was restricted to passages 6–10 for the experiments. To purify BMSCs, the cells were subject to fluorescence-activated cell sorting (FACS) analysis, with collection of cells positive for Sca-1 and CD44 [62], but negative for the hematopoietic stem cell and macrophage marker CD45 [25].

### RNA sample preparation and RNA-seq assay

BMSCs were plated at 1 × 10^5^ cells/well/ml for 24 h and further treated with LPS (200 ng/ml) for another 24 h, and total RNA was extracted before and after LPS treatment, following a standard protocol [25]. Experiments were conducted in triplicate.

Standard methods were used for RNA-seq library construction, EZBead preparation, and Next-Gen sequencing, based on the Life Technologies SOLiD 5500xl system. Briefly, 2 μg of total RNA per sample was used for library preparation. The rRNA was first depleted using the standard protocol of RiboMinus Eukaryote Kit for RNA-Seq (Ambion), and rRNA-depleted RNA was concentrated using a PureLink RNA Micro Kit (Invitrogen) with 1 volume of lysis buffer and 2.5 volumes of 100 % ethanol. After rRNA depletion, a whole transcriptome library was prepared and barcoded per sample using the standard protocol of SOLiD Total RNA-seq Kit (Life Technologies). Each barcoded library was quantified by quantitative polymerase chain reaction (qPCR) using SOLiD Library Taqman qPCR Module (Life Technologies) and pooled in equal molarity. EZBead preparation, bead library amplification, and bead enrichment were then conducted using the Life Technologies EZ Bead E80 System. Finally sequencing by ligation was performed using a standard single-read, 5′-3′ strand-specific sequencing procedure (75 nt-read) on SOLiD 5500xl.

### Bioinformatics analysis for RNA-seq data

RNA-seq data analysis included the following steps: quality assessment, sequence alignment, and alternative splicing analysis. The RNA-seq data can be accessed through the Gene Expression Omnibus (http://www.ncbi.nlm.nih.gov/geo/) with accession number GSE64568).

### Data processing and quality assessment

We used SOLiD Instrument Control Software and SOLiD Experiment Tracking System software for read quality recalibration. Each sequence read was scanned for low-quality regions, and if a 5-base sliding window had an average quality score less than 20, the read was truncated at that position. Any read < 35 bases was discarded. Our experience suggests that this strategy effectively eliminates low-quality reads, while retaining high-quality regions [63–65].

### Sequence alignment

We used BFAST (http://sourceforge.net/projects/bfast/) [66] as our primary alignment algorithm due to its high sensitivity for aligning reads on loci containing small insertions and deletions, as compared to the reference genome (mm9). We then used a TopHat-like strategy [67] to align the sequencing reads containing cross-splicing junctions using NGSUtils (http://ngsutils.org/) [63]. After aligning the reads to a filtering index including repeats, ribosome RNA, and other sequences that were not of interest, we conducted a sequence alignment at three levels: genome, known junctions (University of California Santa Cruz Genome Browser), and novel junctions (based on the enriched regions identified in the genomic alignment). We restricted our analysis to uniquely aligned sequences with no more than two mismatches.

### Alternative splicing analysis

We used MISO (mixture of isoforms) [34] to identify alternatively spliced exons whose splicing patterns were altered after LPS treatment. We first used Samtools (v0.1.19) to merge six RNA-seq samples into two BAM files according to their biological conditions, i.e., control vs. LPS-treated samples. We then estimated Percent Spliced In (PSI or Ψ), which indicates the proportion of RNA isoforms containing the alternatively spliced exon (inclusive isoforms) among all isoforms (inclusive plus exclusive isoforms). We also computed a Bayes factor (BF) to describe the likelihood of an AS event between the LPS-treated and control conditions. A BF of 5 means that an AS event is 5 times more likely to be differentially spliced than not. Both Ψ and BF values were computed by the software package MISO [34]. The difference between Ψ s across the two conditions was defined as ΔΨ. We required each AS event to have a BF > 5 and |ΔΨ| > 0.05 to be considered differentially spliced.

### Ontological annotations

The functions and cellular locations of AS genes were annotated by the pathway analysis tool Ingenuity Pathway Analysis (IPA), and the functional and biochemical properties of these genes were further annotated based on SwissProt and PIR keywords with DAVID v6.7 [38].

### Protein domains overlapping AS regions

Protein domain information was predicted based on the RNA nucleotide sequences of the alternatively spliced exon, and 30-base flanking sequencings of both upstream and downstream exons. These RNA sequences were then translated into peptides, based on open reading frames (ORFs) documented by Ensembl and Refseq, which were then input into Pfam [41] for identification of protein domains overlapping AS regions.

### Identification of protein-protein interactions (PPI)

We also examined whether alternatively spliced exons overlapped with potential protein-protein interaction domains. Based on the protein domains identified in or overlapping AS regions, we retrieved their binding partner domains with iPfam [41], which documents domain-domain interactions in the Protein Data Bank (PDB). We further used Pfam to search for genes encoding partner domains (i.e., potential protein interaction partners). The identified protein interaction partners were verified by two protein-protein interaction databases derived from high-throughput experiments.

### Other characterizations

Protein disorder was predicted with VSL2B [45], a highly regarded protein disorder prediction tool, especially for long regions of disorder [68]. We required the peptides flanking the AS regions to be at least 9 amino acids long for accurate prediction. Potential binding sites were predicted with MoRF2, a software tool that predicts protein-binding sites that undergo a disorder–order transformation while binding another protein molecule [69]. Known post-translational modification (PTM) sites were derived from UniProt, and novel PTM sites were predicted by ModPred [47]. The upstream gene regulator NFYA (Nuclear transcription Factor Y subunit Alpha) [70] was predicted by Ingenuity Pathway Analysis (IPA), based on gene expression data and known regulatory gene interactions.

## Abbreviations

AS, Alternative splicing; BF, Bayesian factor; BMSCs, Bone marrow stromal cells; LPS, Lipopolysaccharide; NMD, Nonsense-mediated decay; PSI, or Ψ, Percent spliced in; PTM, Post-translational modification; PPIs, Protein-protein interactions; UTRs, Untranslated regions

## Additional files

Additional file 1: Statistics of the RNA sequencing experiment. (DOCX 13 kb) Additional file 2: Functions and cellular locations of AS genes. (DOCX 12 kb) Additional file 3: The function and localization of alternatively spliced genes. (DOCX 23 kb) Additional file 4: Alternatively spliced genes containing Molecular Recognition Features (MoRF). (DOCX 12 kb) Additional file 5: Sashimi plot of NFYA. (PDF 121 kb)
