# Supplementary material for: Lipopolysaccharide treatment induces genome-wide pre-mRNA splicing pattern changes in mouse bone marrow stromal stem cells
Source: BMC Genomics. 2016 Aug 22;17(Suppl 7):509. doi: 10.1186/s12864-016-2898-5 (PMC5001229; doi:10.1186/s12864-016-2898-5)
Supplement: Additional file 1: — Statistics of the RNA sequencing experiment. (DOCX 13 kb) [file 12864_2016_2898_MOESM1_ESM.docx]

Additional File 1. Statistics of the RNA sequencing experiment

| **Sample** | **Group** | **Sum of Raw FASTQ** | **Sum of Passed Quality Control (QC) Filter** | **%passQC** | **Sum of Passed Seq Filter (rRNA/tRNA)** | **%passSeqFilter/passQC** | **%passSeqFilter/RawFASTQ** | **Sum of Mapped** | **%mapped/passFilter** | **%Overallmapped** |
| --- | --- | --- | --- | --- | --- | --- | --- | --- | --- | --- |
| Library1_1 | CTR | 56576037 | 50499686 | 89.26 | 38033602 | 75.31 | 67.23 | 31752992 | 83.49 | 56.12 |
| Library2_2 | CTR | 59674412 | 54819003 | 91.86 | 37784090 | 68.93 | 63.32 | 31603786 | 83.64 | 52.96 |
| Library3_3 | CTR | 43434865 | 37622163 | 86.62 | 33262389 | 88.41 | 76.58 | 29070658 | 87.4 | 66.93 |
| Library4_4 | LPS | 47452526 | 42001138 | 88.51 | 35363458 | 84.2 | 74.52 | 30198874 | 85.4 | 63.64 |
| Library5_5 | LPS | 59253111 | 53152924 | 89.7 | 42448510 | 79.86 | 71.64 | 36138497 | 85.13 | 60.99 |
| Library6_6 | LPS | 59897311 | 52868581 | 88.27 | 40708184 | 77 | 67.96 | 34247425 | 84.13 | 57.18 |
