# Supplementary material for: Lipopolysaccharide treatment induces genome-wide pre-mRNA splicing pattern changes in mouse bone marrow stromal stem cells
Source: BMC Genomics. 2016 Aug 22;17(Suppl 7):509. doi: 10.1186/s12864-016-2898-5 (PMC5001229; doi:10.1186/s12864-016-2898-5)
Supplement: Additional file 2: — Functions and cellular locations of AS genes. (DOCX 12 kb) [file 12864_2016_2898_MOESM2_ESM.docx]

Additional File 2. Functions and cellular locations of AS genes

|  | Nucleus | Cytoplasma | Plasma Membrane | Extracellular Space | Other | Total |
| --- | --- | --- | --- | --- | --- | --- |
| Enzyme | 13 | 12 |  |  | 4 | 29 |
| G-protein Coupled Receptor |  |  | 1 |  |  | 1 |
| Transmembrane Receptor |  |  | 3 |  |  | 3 |
| Kinase | 2 | 9 |  |  |  | 11 |
| Ligand-dependent  Nuclear Receptor | 1 |  |  |  |  | 1 |
| Peptidase | 1 | 3 |  |  | 2 | 6 |
| Phosphatase |  |  | 1 |  | 1 | 2 |
| Transcription Regulator | 17 | 2 |  |  | 1 | 20 |
| Transporter |  | 6 | 1 |  |  | 7 |
| Other | 30 | 35 | 12 | 6 | 34 | 117 |
| Total | 64 | 67 | 18 | 6 | 42 | 197 |
