# Supplementary material for: Lipopolysaccharide treatment induces genome-wide pre-mRNA splicing pattern changes in mouse bone marrow stromal stem cells
Source: BMC Genomics. 2016 Aug 22;17(Suppl 7):509. doi: 10.1186/s12864-016-2898-5 (PMC5001229; doi:10.1186/s12864-016-2898-5)
Supplement: Additional file 3: — The function and localization of alternatively spliced genes. (DOCX 23 kb) [file 12864_2016_2898_MOESM3_ESM.docx]

Additional File 3. The function and localization of alternatively spliced genes

| **as_type** | **gene_symbol** | **gene_description** | **gene_location** | **gene_type** | **∆Ψ** |
| --- | --- | --- | --- | --- | --- |
| cassete exon | Mgrn1 | mahogunin ring finger 1, E3 ubiquitin protein ligase | Cytoplasm | enzyme | -0.25 |
| cassete exon | Smox | spermine oxidase | Cytoplasm | enzyme | 0.37 |
| cassete exon | Rhot1 | ras homolog family member T1 | Cytoplasm | enzyme | -0.15 |
| cassete exon | Magi3 | membrane associated guanylate kinase, WW and PDZ domain containing 3 | Cytoplasm | kinase | -0.42 |
| cassete exon | Pank2 | pantothenate kinase 2 | Cytoplasm | kinase | -0.21 |
| cassete exon | Mark3 | MAP/microtubule affinity-regulating kinase 3 | Cytoplasm | kinase | -0.22 |
| cassete exon | Camk1d | calcium/calmodulin-dependent protein kinase ID | Cytoplasm | kinase | -0.31 |
| cassete exon | Cdc42bpa | CDC42 binding protein kinase alpha (DMPK-like) | Cytoplasm | kinase | -0.12 |
| cassete exon | Plscr2 | phospholipid scramblase 2 | Cytoplasm | other | 0.16 |
| cassete exon | Fopnl | FGFR1OP N-terminal like | Cytoplasm | other | 0.11 |
| cassete exon | Cyb561a3 | cytochrome b561 family, member A3 | Cytoplasm | other | 0.37 |
| cassete exon | Mpv17 | MpV17 mitochondrial inner membrane protein | Cytoplasm | other | 0.23 |
| cassete exon | Tbc1d31 | TBC1 domain family, member 31 | Cytoplasm | other | 0.22 |
| cassete exon | Picalm | phosphatidylinositol binding clathrin assembly protein | Cytoplasm | other | -0.06 |
| cassete exon | Numbl | numb homolog (Drosophila)-like | Cytoplasm | other | -0.39 |
| cassete exon | Arhgef11 | Rho guanine nucleotide exchange factor (GEF) 11 | Cytoplasm | other | 0.29 |
| cassete exon | Abi1 | abl-interactor 1 | Cytoplasm | other | 0.14 |
| cassete exon | Spc25 | SPC25, NDC80 kinetochore complex component | Cytoplasm | other | 0.15 |
| cassete exon | Fhl1 | four and a half LIM domains 1 | Cytoplasm | other | -0.05 |
| cassete exon | Mob4 | MOB family member 4, phocein | Cytoplasm | other | 0.25 |
| cassete exon | Spag9 | sperm associated antigen 9 | Cytoplasm | other | 0.2 |
| cassete exon | Mphosph9 | M-phase phosphoprotein 9 | Cytoplasm | other | -0.36 |
| cassete exon | Plec | plectin | Cytoplasm | other | 0.05 |
| cassete exon | Xpnpep3 | X-prolyl aminopeptidase (aminopeptidase P) 3, putative | Cytoplasm | peptidase | 0.27 |
| cassete exon | Blmh | bleomycin hydrolase | Cytoplasm | peptidase | 0.25 |
| cassete exon | Fabp5 | fatty acid binding protein 5 (psoriasis-associated) | Cytoplasm | transporter | 0.1 |
| cassete exon | Copg2 | coatomer protein complex, subunit gamma 2 | Cytoplasm | transporter | 0.2 |
| cassete exon | Rabep1 | rabaptin, RAB GTPase binding effector protein 1 | Cytoplasm | transporter | 0.14 |
| cassete exon | Pctp | phosphatidylcholine transfer protein | Cytoplasm | transporter | 0.39 |
| cassete exon | Arl13b | ADP-ribosylation factor-like 13B | Extracellular Space | other | -0.29 |
| cassete exon | Slit2 | slit homolog 2 (Drosophila) | Extracellular Space | other | 0.53 |
| cassete exon | Baz2b | bromodomain adjacent to zinc finger domain, 2B | Extracellular Space | other | -0.39 |
| cassete exon | Suv420h1 | suppressor of variegation 4-20 homolog 1 (Drosophila) | Nucleus | enzyme | 0.12 |
| cassete exon | Clk4 | CDC-like kinase 4 | Nucleus | kinase | 0.18 |
| cassete exon | Uty | ubiquitously transcribed tetratricopeptide repeat gene, Y chromosome | Nucleus | other | 0.25 |
| cassete exon | Sun1 | Sad1 and UNC84 domain containing 1 | Nucleus | other | -0.21 |
| cassete exon | Maf1 | MAF1 homolog (S. cerevisiae) | Nucleus | other | -0.2 |
| cassete exon | Morf4l2 | mortality factor 4 like 2 | Nucleus | other | 0.17 |
| cassete exon | Hmgxb4 | HMG box domain containing 4 | Nucleus | other | -0.22 |
| cassete exon | Phf20 | PHD finger protein 20 | Nucleus | other | -0.13 |
| cassete exon | Zfp120 | zinc finger protein 932 | Nucleus | other | -0.33 |
| cassete exon | Ift122 | intraflagellar transport 122 homolog (Chlamydomonas) | Nucleus | other | 0.38 |
| cassete exon | Phf7 | PHD finger protein 7 | Nucleus | other | -0.46 |
| cassete exon | Ctnnd1 | catenin (cadherin-associated protein), delta 1 | Nucleus | other | -0.22 |
| cassete exon | Rad18 | RAD18 homolog (S. cerevisiae) | Nucleus | other | 0.23 |
| cassete exon | Senp7 | SUMO1/sentrin specific peptidase 7 | Nucleus | peptidase | -0.52 |
| cassete exon | Depdc1a | DEP domain containing 1 | Nucleus | TR | 0.16 |
| cassete exon | Ybx3 | Y box binding protein 3 | Nucleus | TR | -0.12 |
| cassete exon | Ncor1 | nuclear receptor corepressor 1 | Nucleus | TR | -0.33 |
| cassete exon | Kansl2 | KAT8 regulatory NSL complex subunit 2 | Other | enzyme | 0.22 |
| cassete exon | Ube2q2 | ubiquitin-conjugating enzyme E2Q family member 2 | Other | enzyme | 0.31 |
| cassete exon | Kansl2 | KAT8 regulatory NSL complex subunit 2 | Other | enzyme | 0.22 |
| cassete exon | Rnf214 | ring finger protein 214 | Other | other | 0.37 |
| cassete exon | Tmem161b | transmembrane protein 161B | Other | other | 0.19 |
| cassete exon | Zfp740 | zinc finger protein 740 | Other | other | -0.3 |
| cassete exon | Ubl4a | Slc10a3-Ubl4 readthrough | Other | other | -0.12 |
| cassete exon | Asb7 | ankyrin repeat and SOCS box containing 7 | Other | other | 0.36 |
| cassete exon | Lins | lines homolog (Drosophila) | Other | other | 0.24 |
| cassete exon | Ttc13 | tetratricopeptide repeat domain 13 | Other | other | -0.21 |
| cassete exon | Slx4ip | SLX4 interacting protein | Other | other | -0.3 |
| cassete exon | Ppp4r1l-ps | protein phosphatase 4, regulatory subunit 1-like, pseudogene | Other | other | -0.41 |
| cassete exon | Smim8 | small integral membrane protein 8 | Other | other | 0.27 |
| cassete exon | Zmym4 | zinc finger, MYM-type 4 | Other | other | 0.47 |
| cassete exon | Smim8 | small integral membrane protein 8 | Other | other | 0.27 |
| cassete exon | Svil | supervillin | Other | other | 0.22 |
| cassete exon | Adprm | ADP-ribose/CDP-alcohol diphosphatase, manganese-dependent | Other | other | 0.34 |
| cassete exon | Tbc1d9b | TBC1 domain family, member 9B (with GRAM domain) | Other | other | -0.4 |
| cassete exon | Pwwp2a | PWWP domain containing 2A | Other | other | 0.53 |
| cassete exon | Adprm | ADP-ribose/CDP-alcohol diphosphatase, manganese-dependent | Other | other | 0.34 |
| cassete exon | Smim8 | small integral membrane protein 8 | Other | other | 0.27 |
| cassete exon | 2810474O19Rik | KIAA1551 | Other | other | 0.28 |
| cassete exon | Usp45 | ubiquitin specific peptidase 45 | Other | peptidase | -0.41 |
| cassete exon | Prepl | prolyl endopeptidase-like | Other | peptidase | 0.14 |
| cassete exon | Rnf14 | ring finger protein 14 | Other | TR | -0.19 |
| cassete exon | Tmem11 | transmembrane protein 11 | PM | GPCR | 0.16 |
| cassete exon | Ttll7 | tubulin tyrosine ligase-like family, member 7 | PM | other | 0.38 |
| cassete exon | Aif1l | allograft inflammatory factor 1-like | PM | other | 0.32 |
| cassete exon | Aif1l | allograft inflammatory factor 1-like | PM | other | 0.32 |
| cassete exon | Dnajc5 | DnaJ (Hsp40) homolog, subfamily C, member 5 | PM | other | -0.18 |
| cassete exon | Cpeb4 | cytoplasmic polyadenylation element binding protein 4 | PM | other | 0.52 |
| cassete exon | Tpm1 | tropomyosin 1, alpha | PM | other | -0.05 |
| cassete exon | Jmjd6 | jumonji domain containing 6 | PM | TMR | 0.14 |
| A3SS | Eci2 | enoyl-CoA delta isomerase 2 | Cytoplasm | enzyme | 0.16 |
| A3SS | Cyp4f16 | cytochrome P450, family 4, subfamily f, polypeptide 16 | Cytoplasm | enzyme | -0.49 |
| A3SS | Rabggtb | Rab geranylgeranyltransferase, beta subunit | Cytoplasm | enzyme | 0.09 |
| A3SS | Akt1 | v-akt murine thymoma viral oncogene homolog 1 | Cytoplasm | kinase | 0.29 |
| A3SS | Ppip5k2 | diphosphoinositol pentakisphosphate kinase 2 | Cytoplasm | kinase | 0.26 |
| A3SS | Cdv3 | CDV3 homolog (mouse) | Cytoplasm | other | 0.05 |
| A3SS | Dock9 | dedicator of cytokinesis 9 | Cytoplasm | other | 0.48 |
| A3SS | Akt1s1 | AKT1 substrate 1 (proline-rich) | Cytoplasm | other | 0.08 |
| A3SS | Scoc | short coiled-coil protein | Cytoplasm | other | -0.23 |
| A3SS | Becn1 | beclin 1, autophagy related | Cytoplasm | other | -0.1 |
| A3SS | Blmh | bleomycin hydrolase | Cytoplasm | peptidase | 0.25 |
| A3SS | Rrbp1 | ribosome binding protein 1 | Cytoplasm | transporter | -0.26 |
| A3SS | Arfgap1 | ADP-ribosylation factor GTPase activating protein 1 | Cytoplasm | transporter | -0.4 |
| A3SS | Sparc | secreted protein, acidic, cysteine-rich (osteonectin) | Extracellular Space | other | 0.1 |
| A3SS | Bod1l | biorientation of chromosomes in cell division 1-like 1 | Extracellular Space | other | 0.34 |
| A3SS | Mettl3 | methyltransferase like 3 | Nucleus | enzyme | 0.45 |
| A3SS | Tyms | thymidylate synthetase | Nucleus | enzyme | 0.09 |
| A3SS | Mcm9 | minichromosome maintenance complex component 9 | Nucleus | enzyme | -0.46 |
| A3SS | Rev1 | REV1, polymerase (DNA directed) | Nucleus | enzyme | -0.22 |
| A3SS | Zfp346 | zinc finger protein 346 | Nucleus | other | 0.39 |
| A3SS | Chchd1 | coiled-coil-helix-coiled-coil-helix domain containing 1 | Nucleus | other | -0.12 |
| A3SS | Fra10ac1 | fragile site, folic acid type, rare, fra(10)(q23.3) or fra(10)(q24.2) candidate 1 | Nucleus | other | 0.43 |
| A3SS | Srrt | serrate RNA effector molecule homolog (Arabidopsis) | Nucleus | other | 0.22 |
| A3SS | Ivns1abp | influenza virus NS1A binding protein | Nucleus | other | 0.07 |
| A3SS | Hnrnpr | heterogeneous nuclear ribonucleoprotein R | Nucleus | other | 0.18 |
| A3SS | Cdc27 | cell division cycle 27 | Nucleus | other | -0.2 |
| A3SS | Phrf1 | PHD and ring finger domains 1 | Nucleus | other | -0.33 |
| A3SS | Nolc1 | nucleolar and coiled-body phosphoprotein 1 | Nucleus | TR | 0.14 |
| A3SS | Htatip2 | HIV-1 Tat interactive protein 2, 30kDa | Nucleus | TR | 0.4 |
| A3SS | Rbm39 | RNA binding motif protein 39 | Nucleus | TR | -0.21 |
| A3SS | Hdac10 | histone deacetylase 10 | Nucleus | TR | -0.44 |
| A3SS | Nfya | nuclear transcription factor Y, alpha | Nucleus | TR | -0.32 |
| A3SS | Ankzf1 | ankyrin repeat and zinc finger domain containing 1 | Nucleus | TR | -0.42 |
| A3SS | Fus | fused in sarcoma | Nucleus | TR | 0.21 |
| A3SS | Sbno1 | strawberry notch homolog 1 (Drosophila) | Other | enzyme | 0.3 |
| A3SS | 4833420G17Rik | chromosome 5 open reading frame 34 | Other | other | -0.38 |
| A3SS | Eml3 | echinoderm microtubule associated protein like 3 | Other | other | -0.42 |
| A3SS | Phf20l1 | PHD finger protein 20-like 1 | Other | other | -0.27 |
| A3SS | Ubl4a | Slc10a3-Ubl4 readthrough | Other | other | -0.12 |
| A3SS | Dda1 | DET1 and DDB1 associated 1 | Other | other | 0.07 |
| A3SS | Ttpal | tocopherol (alpha) transfer protein-like | Other | other | 0.41 |
| A3SS | Miip | migration and invasion inhibitory protein | Other | other | 0.18 |
| A3SS | Ppp4r1 | protein phosphatase 4, regulatory subunit 1 | Other | phosphatase | 0.23 |
| A3SS | Ly6a | lymphocyte antigen 6 complex, locus A | PM | other | 0.44 |
| A3SS | Prnd | prion protein 2 (dublet) | PM | other | -0.45 |
| A5SS | Ndufs1 | NADH dehydrogenase (ubiquinone) Fe-S protein 1, 75kDa (NADH-coenzyme Q reductase) | Cytoplasm | enzyme | -0.24 |
| A5SS | Birc6 | baculoviral IAP repeat containing 6 | Cytoplasm | enzyme | 0.28 |
| A5SS | Nit1 | nitrilase 1 | Cytoplasm | enzyme | -0.33 |
| A5SS | Map3k7 | mitogen-activated protein kinase kinase kinase 7 | Cytoplasm | kinase | -0.4 |
| A5SS | Ehbp1l1 | EH domain binding protein 1-like 1 | Cytoplasm | other | -0.05 |
| A5SS | Cmc2 | COX assembly mitochondrial protein 2 homolog (S. cerevisiae) | Cytoplasm | other | 0.1 |
| A5SS | Spc25 | SPC25, NDC80 kinetochore complex component | Cytoplasm | other | 0.15 |
| A5SS | Dnajb12 | DnaJ (Hsp40) homolog, subfamily B, member 12 | Cytoplasm | other | -0.52 |
| A5SS | Mrps33 | mitochondrial ribosomal protein S33 | Cytoplasm | other | -0.22 |
| A5SS | Mkks | McKusick-Kaufman syndrome | Cytoplasm | other | -0.17 |
| A5SS | Ddx6 | DEAD (Asp-Glu-Ala-Asp) box helicase 6 | Nucleus | enzyme | 0.14 |
| A5SS | Fen1 | flap structure-specific endonuclease 1 | Nucleus | enzyme | 0.1 |
| A5SS | Ddx6 | DEAD (Asp-Glu-Ala-Asp) box helicase 6 | Nucleus | enzyme | 0.14 |
| A5SS | Naa10 | N(alpha)-acetyltransferase 10, NatA catalytic subunit | Nucleus | enzyme | 0.28 |
| A5SS | Adarb1 | adenosine deaminase, RNA-specific, B1 | Nucleus | enzyme | 0.23 |
| A5SS | Nr1h2 | nuclear receptor subfamily 1, group H, member 2 | Nucleus | ligand-dependent nuclear receptor | 0.29 |
| A5SS | Srsf7 | serine/arginine-rich splicing factor 7 | Nucleus | other | 0.19 |
| A5SS | Srsf7 | serine/arginine-rich splicing factor 7 | Nucleus | other | 0.19 |
| A5SS | Zfp60 | zinc finger protein 60 | Nucleus | other | -0.29 |
| A5SS | Hira | histone cell cycle regulator | Nucleus | TR | 0.15 |
| A5SS | Yeats2 | YEATS domain containing 2 | Nucleus | TR | -0.47 |
| A5SS | Tmem234 | transmembrane protein 234 | Other | other | 0.31 |
| A5SS | Arl16 | ADP-ribosylation factor-like 16 | Other | other | -0.3 |
| A5SS | Zfp740 | zinc finger protein 740 | Other | other | -0.3 |
| A5SS | Efr3a | EFR3 homolog A (S. cerevisiae) | PM | other | -0.1 |
| A5SS | Smek1 | SMEK homolog 1, suppressor of mek1 (Dictyostelium) | PM | other | 0.22 |
| A5SS | Inpp5a | inositol polyphosphate-5-phosphatase, 40kDa | PM | phosphatase | 0.2 |
| A5SS | Jmjd6 | jumonji domain containing 6 | PM | TMR | 0.14 |
| retained intron | Hspa8 | heat shock 70kDa protein 8 | Cytoplasm | enzyme | 0.12 |
| retained intron | Trim2 | tripartite motif containing 2 | Cytoplasm | enzyme | 0.17 |
| retained intron | Coq6 | coenzyme Q6 monooxygenase | Cytoplasm | enzyme | 0.38 |
| retained intron | Dgkq | diacylglycerol kinase, theta 110kDa | Cytoplasm | kinase | 0.29 |
| retained intron | Yipf2 | Yip1 domain family, member 2 | Cytoplasm | other | 0.32 |
| retained intron | Rasa2 | RAS p21 protein activator 2 | Cytoplasm | other | 0.24 |
| retained intron | Wipi2 | WD repeat domain, phosphoinositide interacting 2 | Cytoplasm | other | 0.19 |
| retained intron | Rps18 | ribosomal protein S18 | Cytoplasm | other | 0.09 |
| retained intron | Wdr11 | WD repeat domain 11 | Cytoplasm | other | -0.21 |
| retained intron | Golga1 | golgin A1 | Cytoplasm | other | 0.15 |
| retained intron | Becn1 | beclin 1, autophagy related | Cytoplasm | other | -0.1 |
| retained intron | Lrrc45 | leucine rich repeat containing 45 | Cytoplasm | other | -0.28 |
| retained intron | Ambra1 | autophagy/beclin-1 regulator 1 | Cytoplasm | other | -0.37 |
| retained intron | Eif4a2 | eukaryotic translation initiation factor 4A2 | Cytoplasm | TLR | 0.09 |
| retained intron | Eif4a2 | eukaryotic translation initiation factor 4A2 | Cytoplasm | TLR | 0.09 |
| retained intron | Tmem214 | transmembrane protein 214 | Extracellular Space | other | -0.4 |
| retained intron | Msh3 | mutS homolog 3 | Nucleus | enzyme | 0.31 |
| retained intron | Nle1 | notchless homolog 1 (Drosophila) | Nucleus | enzyme | 0.39 |
| retained intron | Sirt7 | sirtuin 7 | Nucleus | enzyme | 0.27 |
| retained intron | Nek8 | NIMA-related kinase 8 | Nucleus | kinase | -0.38 |
| retained intron | Mis18a | MIS18 kinetochore protein homolog A (S. pombe) | Nucleus | other | 0.16 |
| retained intron | 9930012K11Rik | chromosome 8 open reading frame 58 | Nucleus | other | 0.25 |
| retained intron | Ncaph2 | non-SMC condensin II complex, subunit H2 | Nucleus | other | -0.08 |
| retained intron | Lrif1 | ligand dependent nuclear receptor interacting factor 1 | Nucleus | other | 0.28 |
| retained intron | Xab2 | XPA binding protein 2 | Nucleus | other | 0.23 |
| retained intron | Sapcd2 | suppressor APC domain containing 2 | Nucleus | other | 0.39 |
| retained intron | Cdan1 | codanin 1 | Nucleus | other | 0.34 |
| retained intron | Hnrnph1 | heterogeneous nuclear ribonucleoprotein H1 (H) | Nucleus | other | -0.06 |
| retained intron | Gtf2a2 | general transcription factor IIA, 2, 12kDa | Nucleus | TR | 0.28 |
| retained intron | Hsf1 | heat shock transcription factor 1 | Nucleus | TR | -0.34 |
| retained intron | Tfe3 | transcription factor binding to IGHM enhancer 3 | Nucleus | TR | 0.16 |
| retained intron | Trim28 | tripartite motif containing 28 | Nucleus | TR | 0.25 |
| retained intron | Snapc4 | small nuclear RNA activating complex, polypeptide 4, 190kDa | Nucleus | TR | -0.31 |
| retained intron | Mroh1 | maestro heat-like repeat family member 1 | Other | other | 0.2 |
| retained intron | Morc4 | MORC family CW-type zinc finger 4 | Other | other | -0.3 |
| retained intron | Dus1l | dihydrouridine synthase 1-like (S. cerevisiae) | Other | other | 0.15 |
| retained intron | Slc7a6os | solute carrier family 7, member 6 opposite strand | Other | other | -0.14 |
| retained intron | Psmd5 | proteasome (prosome, macropain) 26S subunit, non-ATPase, 5 | Other | other | -0.06 |
| retained intron | Sharpin | SHANK-associated RH domain interactor | PM | other | 0.22 |
| retained intron | Lmbr1l | limb development membrane protein 1-like | PM | other | -0.4 |
| retained intron | Jmjd6 | jumonji domain containing 6 | PM | TMR | 0.14 |
| retained intron | Slc39a7 | solute carrier family 39 (zinc transporter), member 7 | PM | transporter | -0.1 |

TR: transcription regulator

TMR: transmembrane receptor

TLR: translation regulator

GPCR: G-protein coupled receptor

PM: plasma membrane
