# Supplementary material for: Lipopolysaccharide treatment induces genome-wide pre-mRNA splicing pattern changes in mouse bone marrow stromal stem cells
Source: BMC Genomics. 2016 Aug 22;17(Suppl 7):509. doi: 10.1186/s12864-016-2898-5 (PMC5001229; doi:10.1186/s12864-016-2898-5)
Supplement: Additional file 4: — Alternatively spliced genes containing Molecular Recognition Features (MoRF). (DOCX 12 kb) [file 12864_2016_2898_MOESM4_ESM.docx]

Additional File 4. Alternatively spliced genes containing Molecular Recognition Features (MoRF)

| **gene_symbol** | **gene_description** |
| --- | --- |
| Arl13b | ADP-ribosylation factor-like 13B |
| Senp7 | SUMO1/sentrin specific peptidase 7 |
| Tbc1d31 | TBC1 domain family, member 31 |
| Depdc1a | DEP domain containing 1 |
| Arhgef11 | Rho guanine nucleotide exchange factor (GEF) 11 |
| Aif1l | allograft inflammatory factor 1-like |
| Abi1 | abl-interactor 1 |
| Usp45 | ubiquitin specific peptidase 45 |
| Ybx3 | Y box binding protein 3 |
| Cpeb4 | cytoplasmic polyadenylation element binding protein 4 |
| Rabep1 | rabaptin, RAB GTPase binding effector protein 1 |
| Ncor1 | nuclear receptor corepressor 1 |
| Ube2q2 | ubiquitin-conjugating enzyme E2Q family member 2 |
| Ctnnd1 | catenin (cadherin-associated protein), delta 1 |
| Plec | plectin |
| Cdc42bpa | CDC42 binding protein kinase alpha (DMPK-like) |
| Ambra1 | autophagy/beclin-1 regulator 1 |
| Ehbp1l1 | EH domain binding protein 1-like 1 |
| Zfp346 | zinc finger protein 346 |
| Nolc1 | nucleolar and coiled-body phosphoprotein 1 |
| Rrbp1 | ribosome binding protein 1 |
| Miip | migration and invasion inhibitory protein |
