# Supplementary figures and images for: Lipopolysaccharide treatment induces genome-wide pre-mRNA splicing pattern changes in mouse bone marrow stromal stem cells
Source: BMC Genomics. 2016 Aug 22;17(Suppl 7):509. doi: 10.1186/s12864-016-2898-5 (PMC5001229; doi:10.1186/s12864-016-2898-5)

Nfya  $\Delta\Psi=-0.31$

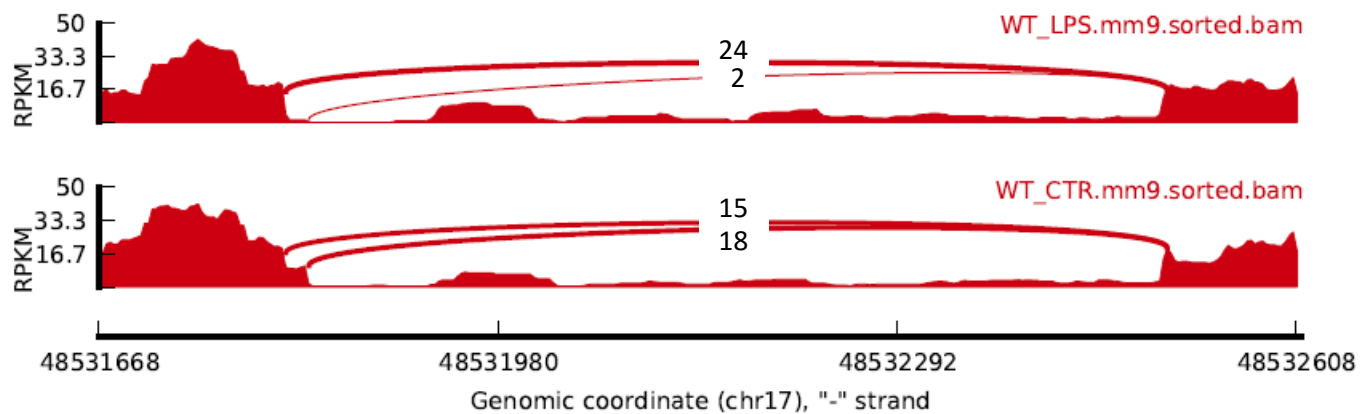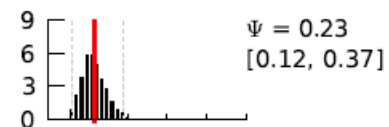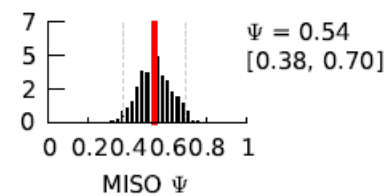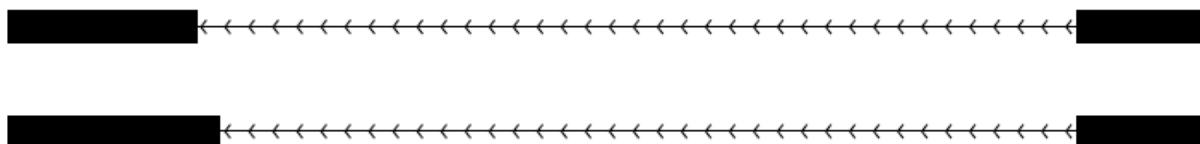

Supplement: Additional file 5: — Sashimi plot of NFYA. (PDF 121 kb) [file 12864_2016_2898_MOESM5_ESM.pdf]
